# Multi-pinhole fluorescent x-ray computed tomography for molecular imaging

**DOI:** 10.1038/s41598-017-05179-2

**Published:** 2017-07-18

**Authors:** Tenta Sasaya, Naoki Sunaguchi, Kazuyuki Hyodo, Tsutomu Zeniya, Tetsuya Yuasa

**Affiliations:** 1 Graduate School of Science and Engineering, Yamagata University, Yonezawa, Japan; 2 Graduate School of Medicine, Nagoya University, Nagoya, Japan; 3 Institute of Materials Structure Science, High Energy Accelerator Organization (KEK), Tsukuba, Japan; 4 Graduate School of Science and Technology, Hirosaki University, Hirosaki, Japan

## Abstract

We propose a multi-pinhole fluorescent x-ray computed tomography (mp-FXCT) technique for preclinical molecular imaging that can provide the complete data necessary to produce 3-D tomographic images during anaesthesia. In this method, multiple projections are simultaneously acquired through a multi-pinhole collimator with a 2-D detector and full-field volumetric beam to accelerate the data acquisition process and enhance the signal-to-noise ratios of the projections. We constructed a 15-pinhole mp-FXCT imaging system at beamline ARNE-7A at KEK and performed preliminary experiments to investigate its imaging properties using physical phantoms and a non-radioactive I imaging agent. The mp-FXCT system could detect an I concentration of 0.038 mg/ml, the minimum required for *in-vivo* imaging, at a spatial resolution of about 0.3 mm during a data acquisition time of 90 min, which is less than the time for which anaesthesia is effective and suggests that preclinical molecular imaging is feasible with mp-FXCT.

## Introduction

Recent advances in genetic and tissue engineering have yielded rodent models of human diseases that provide important clues regarding their causes, diagnoses, and treatment. Consequently, studies using mice and rats have become important in areas such as molecular biology, toxicology, and drug discovery research. Hypotheses regarding the onset of diseases and the effectiveness of treatment can be tested using animals before performing studies on humans. Furthermore, genome manipulation and transplantation of carcinoma cells differentiated from stem cells can be performed using rodents, enhancing the understanding of human diseases. Molecular imaging techniques such as positron emission tomography and single-photon emission computed tomography (SPECT) are important for observing physiological and pathological characteristics *in vivo* ^1–3^. However, their spatial resolutions remain insufficient. In addition, they require radioactive imaging agents, resulting in measurement difficulty. Thus, a novel, high-contrast, high-spatial-resolution molecular imaging technique using non-radioactive imaging agents would be advantageous.

X-ray fluorescence (XRF) analysis is a highly sensitive physicochemical method that enables quantitative element identification by collecting fluorescent x-ray photons emitted by the element of interest. Due to the high brilliance and collimation of synchrotron x-rays, their use as sources further increases the sensitivity of XRF analysis^4^. Fluorescent x-ray computed tomography (FXCT) (also known as XRF computed tomography) using synchrotron x-ray sources has been developed based on the outstanding abilities of XRF analysis. It can be used to delineate the spatial distributions of trace elements in a living body with high sensitivity and has many applications in the material and biomedical sciences^5–15^. Takeda *et al*. demonstrated that FXCT could potentially be used as a small-animal molecular imaging modality, *i.e*., a preclinical molecular imaging modality, by applying FXCT to *in-vivo* imaging of a mouse brain using a non-radioactive I-labelled imaging agent, iodoamphetamine analogue (^127^I-IMP), to delineate *in-vivo* cerebral perfusion with 0.5 mm in-plane spatial resolution^16^. These *in-vivo* FXCT images were obtained using an FXCT system based on first-generation computed tomography, by acquiring sets of projections using a high-energy-resolution detector and thin monochromatic parallel beam^17, 18^. Although FXCT enables highly sensitive detection and provides a spatial resolution of hundreds of micrometres, it is hindered by the long measurement time, which is required because the projection data are acquired from sequential translational and rotational scans of a thin beam, comparable in size to the resolution. Therefore, both the *in-vivo* and *ex-vivo* FXCT images of biomedical samples reported thus far have been single-cross-sectional. For 3-D imaging, a 2-D projection batch acquisition scheme is indispensable^19, 20^.

We recently proposed a pinhole-based FXCT (p-FXCT) system with a 2-D detector and volumetric beam^21^, which enabled faster data acquisition by completely eliminating the need for translational scans and allowed the first 3-D *ex-vivo* FXCT image of a biological sample to be obtained^22^. However, the projections used for reconstruction in p-FXCT are acquired by using a pinhole to limit the directions from which the fluorescent x-ray photons emitted by an object can reach the detector. Inevitably, the number of fluorescence photons acquired by the detector is constrained, increasing the data acquisition time required to obtain projections with sufficient signal-to-noise ratios and deteriorating the imaging properties such as the limit of detection (LOD) and spatial resolution.

To enhance the fluorescence photon acquisition, we developed a multi-pinhole FXCT (mp-FXCT) system in this study. Multi-pinhole collimators have also been used in SPECT to improve the signal-to-noise ratios of projections^23, 24^. Furthermore, we demonstrated the superiority of mp-FXCT to single-pinhole FXCT, and our preliminary experimental results prove its usability for preclinical molecular imaging. In this report, we describe the imaging geometry, imaging principle, and multi-pinhole collimator design. We then specify the imaging properties of an mp-FXCT system constructed at beamline ARNE-7A at KEK and describe the preliminary experiments conducted using physical phantoms. Finally, we discuss the usability of mp-FXCT for preclinical molecular imaging and describe the areas to be improved.

## Method

### mp-FXCT setup

**Figure 1:**
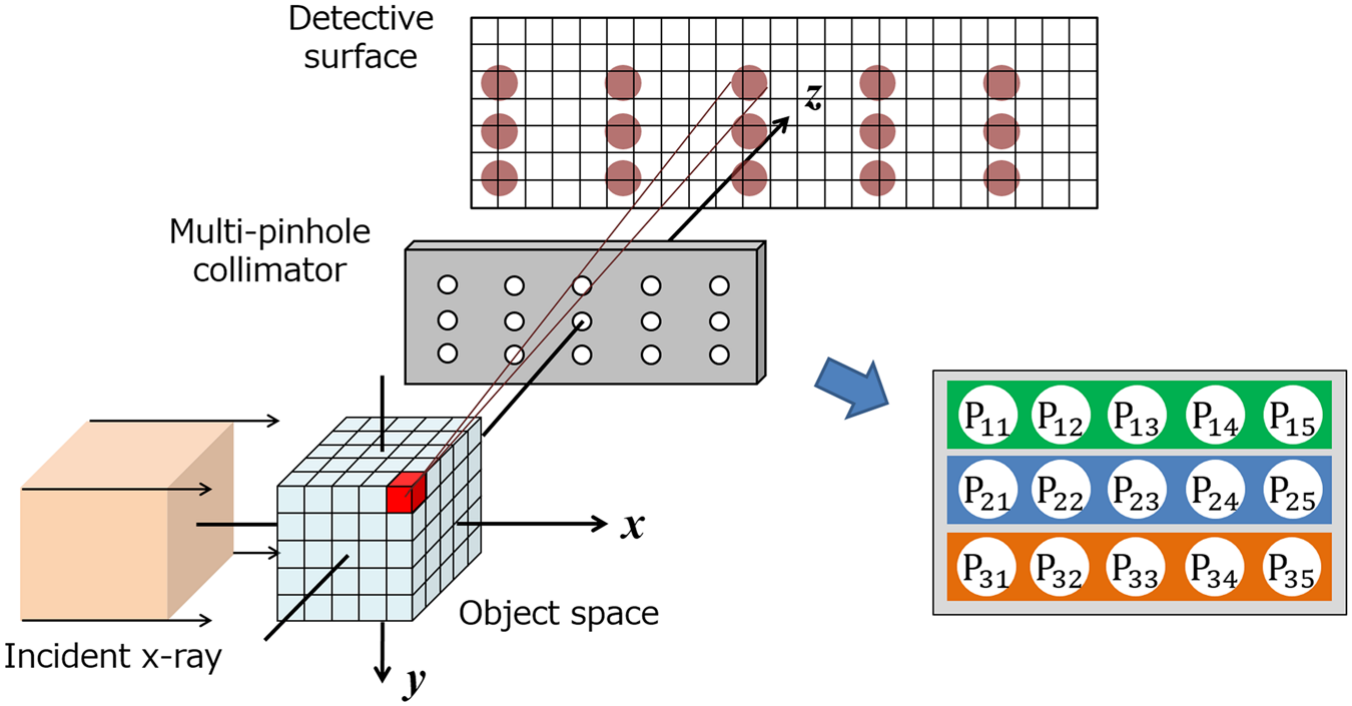
Schematic of the mp-FXCT imaging geometry and pinholes.

The proposed mp-FXCT imaging system consists of a rotational stage for object positioning, a multi-pinhole collimator, an a 2-D detector with multiple elements whose energy resolution was not sufficiently high to enable discrimination between the fluorescent and stray scattered x-rays according to their energies (Pilatus 100 K operating in photon counting mode, manufactured by Dectris). Figure 1 depicts the geometry of the mp-FXCT system in the *xyz* coordinate system. A monochromatic parallel incident beam propagates along the *x*-axis, and the surfaces of the multi-pinhole collimator and 2-D detector are perpendicular to the *z*-axis. The volumetric incident beam irradiates an object near the origin and entirely covers the object. Imaging agents in the object are excited by the incident beam and isotropically emit fluorescent x-ray photons on de-excitation. The emitted fluorescent x-ray photons passing through the pinholes are acquired by the detector. Thus, each view includes multiple projections. The data acquisition process is repeated while rotating the object around the *y*-axis.

### Multi-pinhole collimator design

**Figure 2:**
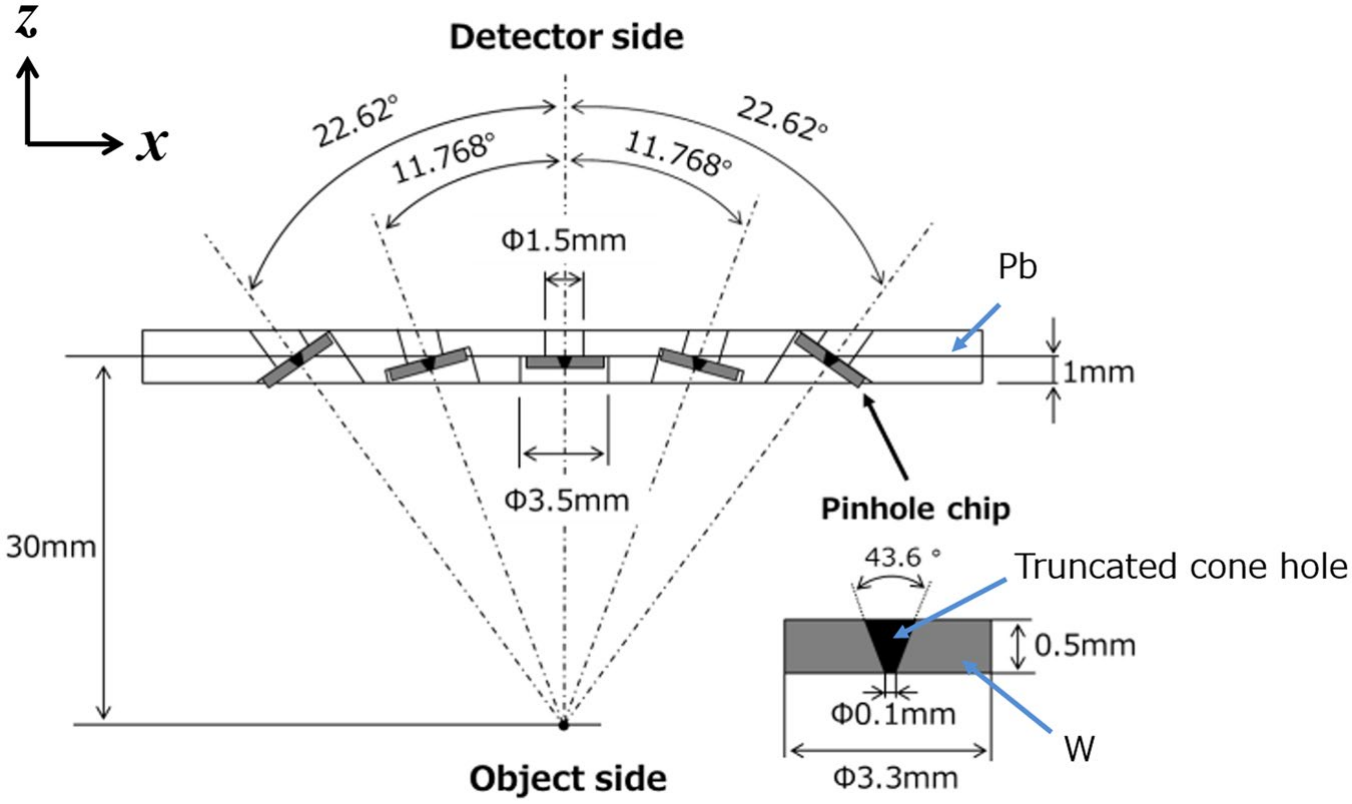
Cross-section of the Pb plate containing the centres of the pinholes and a W pinhole tip.

**Figure 3:**
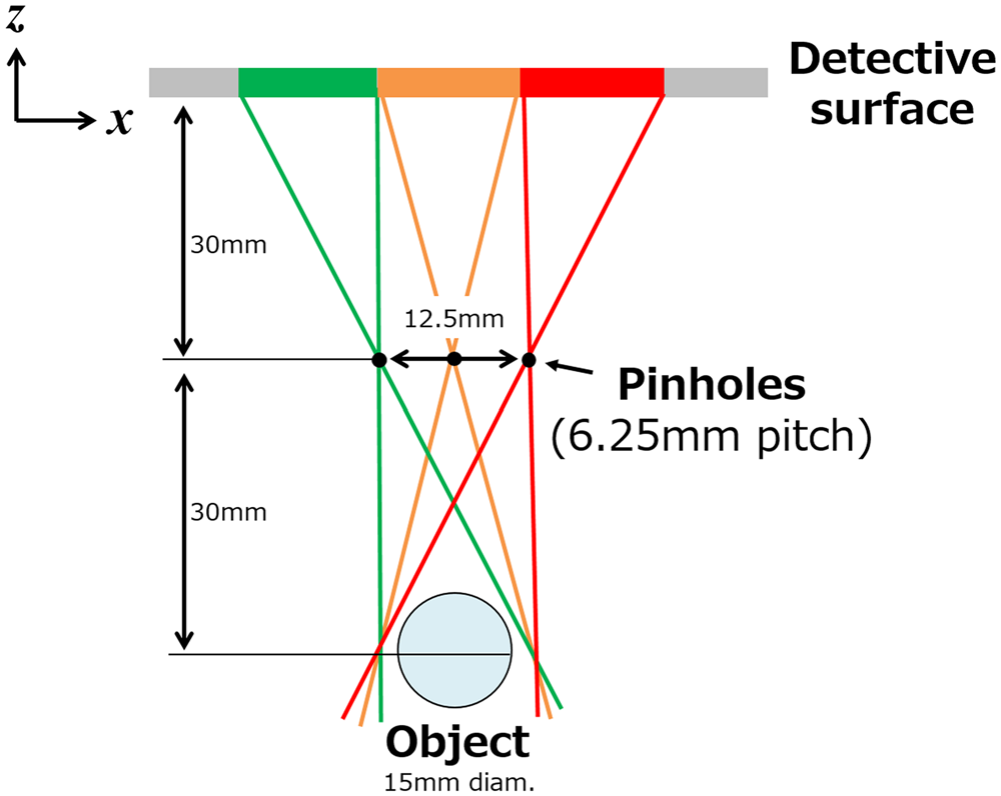
Top view of the geometric arrangement relation of an object, the multi-pinhole collimator, and the detector.

We fabricated a multi-pinhole collimator prototype with 15 pinholes aligned in three rows containing five pinholes each and labelled as shown in Fig. 1. The collimator consisted of a 2-mm-thick Pb (HPbP_4_) plate and 15 W pinhole tips. Figure 2 depicts a cross-section of the Pb plate containing the centres of the pinholes and a W pinhole tip. Fifteen 1.5-mm-diameter holes were bored into the Pb plate so that their central axes were focused at a point 30 mm from the surface of the plate on the object side. Each hole had a 3.5-mm-diameter counterbore to enable the installation of a 3.3-mm-diameter W pinhole tip with a truncated-cone-like hole having a 0.1-mm-diameter upper base on the object side and an opening angle of 43.6°. The centres of the adjacent holes were separated by 6.25 mm so that the projections acquired 30 mm from the collimator plate would not overlap, as shown in Fig. 3.

The entire collimator surface was covered with a layer of 0.15-mm-thick Sn, whose K-edge absorption energy is 29.2 keV. The photoelectric absorption coefficient behaves discontinuously at the K-edge: it suddenly increases just above the K-edge and monotonically decreases below the K-edge. Meanwhile, the Kα-fluorescence energy of I is 28.3 keV. In our situation, in which the incident energy was 33.4 keV, many scattered photons, in addition to fluorescent x-ray photons, had energies less than 33.4 keV. Thus, undesirable scattered photons passed through the pinholes and reached the detector. Since the detector could not differentiate fluorescent photons from scattered photons, the measured data were contaminated, and the reconstructed image quality was degraded. Although Sn would ideally intercept scattered photons with energies greater than 29.2 keV but pass fluorescent 28.3 keV photons, the filtering effect was not complete. We will discuss this point later.

### Formulation

Since the measurement process and formulation were described in detail in ref. 22 for the single-pinhole case, the following discussion starts from the results derived therein. We assume that the object is fixed to the *xyz* coordinate system throughout the projection acquisition process and that the incident beam, pinhole collimator, and detective surface are rotated around the *y*-axis, with their positional relationship maintained, although the object is rotated in the actual measurement process. The index *j* (=1, 2, …, *N*) represents the voxel number, where *N* is the total number of voxels, and *i* (=1, 2, …, *M*) designates the position of the individual detective element, where the detective element positions are consecutively numbered and *M* is the total number of positions. In this situation, the following relationship can be employed:

1 $${y}_{i}=\sum _{j}{p}_{ij}{\lambda }_{j}\quad \quad ({\rm{i}}=1,2,\ldots ,{M}),$$

where *γ*_*i*_, *λ*_*j*_, and *p*_*ij*_ are the number of the fluorescence photons counted at the *i*th position, the I concentration at the *j*th voxel, and the constant of proportionality relating the I concentration at the *j*th voxel to the detected count at the *i*th position, respectively (see Eq. (4) in ref. 22). *p*_*ij*_ can be regarded as the conditional probability that *γ*_*i*_ photons are counted at the *i*th detective position when an I concentration of *λ*_*j*_ exists at the *j*th voxel and is given by the product of the attenuation by the object, incident beam flux, photoelectric linear attenuation coefficient of I, fluorescent yield of I, exposure time, and solid angle subtended by the *j*th voxel through the pinhole, which can be calculated based on the geometry^22^. *γ*_*i*_ and *p*_*ij*_ are known, and *λ*_*j*_ are unknown.

Since a multi-pinhole collimator produces projections for the individual pinholes, Eq. (1) can be rewritten as

2 $${{y}^{k}}_{i}=\sum _{j}{{p}^{k}}_{ij}{{\lambda }^{k}}_{j}\quad \quad ({\rm{i}}=1,2,\ldots ,{M};{k}=1,2,\ldots ,L),$$

where the index *k* is used to identify the pinholes and *L* is the number of pinholes. As a result, we obtain a system of *M* × *L* linear equations and, subsequently, *L* matrix equations ${{\bf{y}}}^{k}={{\bf{P}}}^{k}\,{\boldsymbol{\lambda }}$ ($1\le k\le L$), where ${{\bf{y}}}^{k}={({y}_{1}^{k},{y}_{2}^{k},\ldots ,{y}_{M}^{k})}^{T}$, ${\boldsymbol{\lambda }}={({\lambda }_{1},{\lambda }_{2},\ldots ,{\lambda }_{N})}^{T}$, and

$${{\bf{P}}}^{k}=(\begin{array}{cccc}{p}_{11}^{k} & {p}_{12}^{k} & \cdots  & {p}_{1N}^{k}\\ {p}_{21}^{k} & \ddots  &  & {p}_{2N}^{k}\\ \vdots  &  & \ddots  & \vdots \\ {p}_{M1}^{k} & {p}_{M2}^{k} & \cdots  & {p}_{MN}^{k}\end{array}).$$

In addition, we introduce a *K* (=*L* × *M*)-dimensional vector **f** and a *K* × *N* matrix **E**, where

$$\begin{array}{rcl}{\bf{f}} & = & {({f}_{1},{f}_{2},\cdots ,{f}_{K})}^{T}\\  & = & {({y}_{1}^{1},{y}_{2}^{1},\cdots ,{y}_{M}^{1},{y}_{1}^{2},{y}_{2}^{2},\mathrm{...},{y}_{M}^{2},\cdots ,{y}_{1}^{L},{y}_{2}^{L},\mathrm{...},{y}_{M}^{L})}^{T}\end{array},$$

and

$${\bf{E}}=(\begin{array}{cccc}{e}_{11} & {e}_{12} &  & {e}_{1N}\\ {e}_{21} & \ddots  &  & \\ \vdots  &  & \ddots  & \\ {e}_{K1} & \cdots  &  & {e}_{KN}\end{array})=(\begin{array}{c}{{\bf{P}}}^{1}\\ {{\bf{P}}}^{2}\\ \vdots \\ {{\bf{P}}}^{L}\end{array}).$$

Thus, we obtain the linear inverse problem

3 $${\bf{f}}={\bf{E}}{\boldsymbol{\lambda }},$$

where **f** and **E** are known and **λ** is to be estimated. For reconstruction, we applied the maximum likelihood–expectation maximization (ML-EM) algorithm to solve Eq. (3)^25, 26^.

## Experiment

### Imaging system and calibration

**Figure 4:**
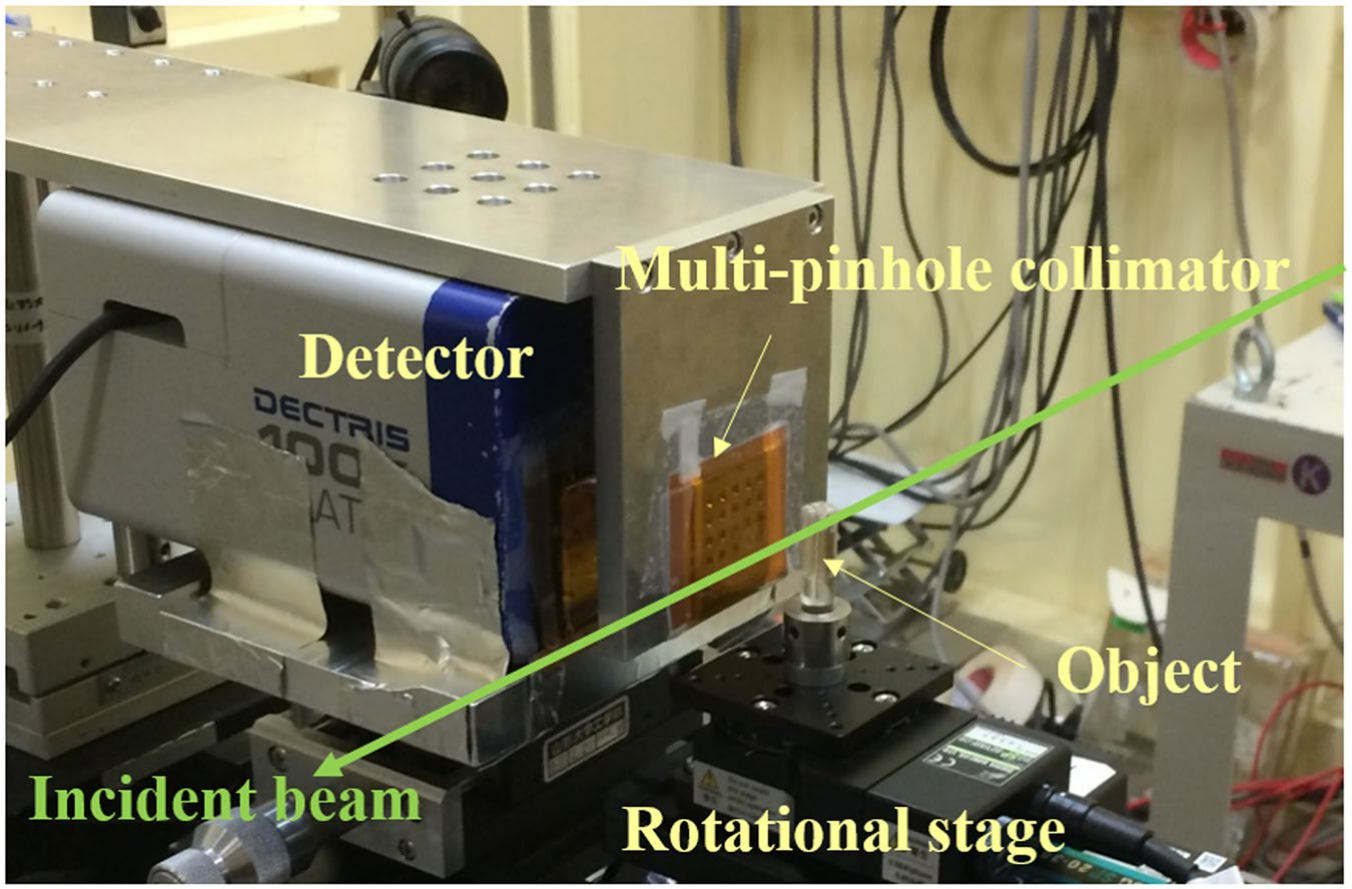
Photograph of the mp-FXCT constructed at beamline ARNE-7A at KEK.

We set up an mp-FXCT imaging system using beamline AR-NE7A (6.5 GeV) at KEK (Fig. 4). The imaging system consisted of a double-crystal Bragg–Bragg monochromator using Si(111) single crystals, an x-ray shutter and slit system, a rotational stage for object positioning, a multi-pinhole collimator, and a 2-D detector. The monochromator was used to select an incident energy of 33.4 keV, just above the 33.17 keV K-edge energy of I, to produce as many Kα fluorescent photons as possible. The flux rate was about 9.3 × 10^7^ photons/mm^2^/s in front of the object. The cross-section of the incident beam was collimated to 35 mm (horizontal) × 5 mm (vertical) by the slit. The actual distances between the rotational axis and collimator plane and between the collimator plane and detector surface were 29.0 mm and 30.6 mm, respectively, while both were designed to be 30.0 mm. A Pilatus 100 K detector (DECTRIS Ltd.) operating in photon counting mode with 487 × 195 elements (pixel size: 172 × 172 μm^2^) and an energy resolution insufficient to discriminate 28.3 keV Kα fluorescence was selected despite its insufficient sensitivity around 30 keV, because it provides a high signal-to-noise ratio as there is no dark current and offers a data transmission rate higher than those of other 2-D detectors^27^. The rotational stage and detector were controlled by a PC. For each object, 90 projections were obtained in 4° increments over 360°.

To realize satisfactory reconstruction, it is essential to calculate *p*_*ij*_ precisely for all *i* and *j*. In particular, the correspondence between the coordinates of the *i*th detective position in each projection image and the *j*th voxel in the 3-D object space should be known as precisely as possible. For this purpose, we prepared a 1-mm-diameter ball containing I solution with a concentration of 10.0 mg/ml. We then related the coordinates of the centre of the ball in the 3-D object space to those of the centre in a projection image and repeated the procedure while changing the position of the ball, which was fixed to the stage. Based on the results, we calculated all of the entries in **E** using Eq. (4) from ref. 22.

### Physical phantoms

**Figure 5:**
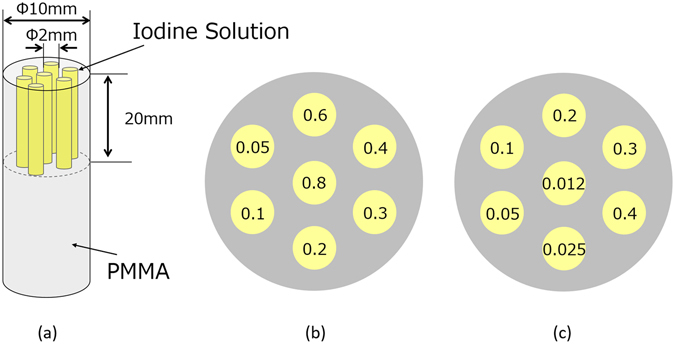
(**a**) Side view of a 10-mm-diameter acrylic cylinder with seven 2-mm-diameter channels filled with I solutions; cross-sections of (**b**) Phantom (I) for verifying the constructed mp-FXCT with concentrations of 0.05, 0.1, 0.2, 0.3, 0.4, 0.6, and 0.8 mg/ml and (**c**) Phantom (II) for evaluating the quantifiability and LOD with concentrations of 0.012, 0.025, 0.05, 0.1, 0.2, 0.3, and 0.4 mg/ml.

Four kinds of physical phantoms were prepared to evaluate the mp-FXCT imaging properties. The first phantom (Phantom (I)) consisted of a 10-mm-diameter PMMA cylinder with seven 2-mm-diameter channels filled with I solutions with different concentrations (0.05, 0.1, 0.2, 0.3, 0.4, 0.6, and 0.8 mg/ml), as shown in Fig. 5(a) and (b). Concentrations higher than those used in *in-vivo* measurements were employed to determine whether the constructed mp-FXCT system operated properly. To investigate the effects of the single-view exposure time on the reconstructed image quality, Phantom (I) was imaged twice, using exposure times of 1 min and 4 min.

The second phantom (Phantom (II)) was utilized to evaluate the LOD and quantifiability. Its container was the same as that of Phantom (I), while the I solutions were replaced with ones of lower concentrations (0.012, 0.025, 0.05, 0.1, 0.2, 0.3, and 0.4 mg/ml), as shown in Fig. 5(c). The exposure time was 1 min.

**Figure 6:**
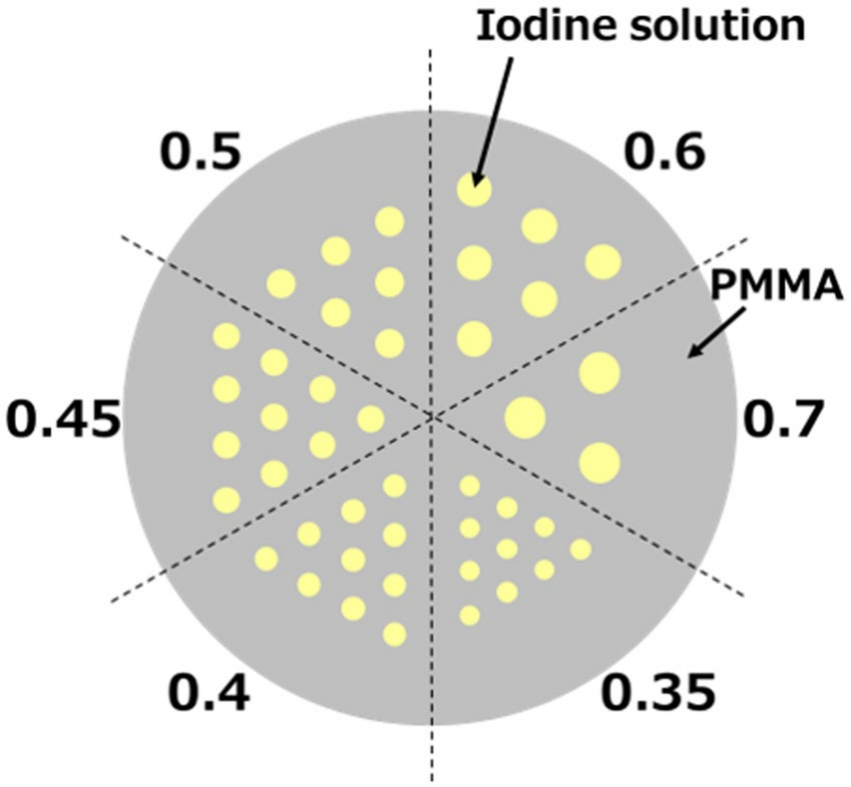
Physical phantom for spatial resolution evaluation (Phantom (III)), consisting of a 10-mm-diameter acrylic cylinder and seven channels of different diameters filled with I solution.

The third phantom (Phantom (III)) was a Derenzo phantom^28^ in which the distance between the adjacent channels was equal to the channel diameter. It was used to evaluate the spatial resolution in a horizontal plane. Channels with six different diameters (0.35, 0.4, 0.45, 0.5, 0.6, and 0.7 mm) were bored into a 10-mm-diameter PMMA cylinder (Fig. 6), and each channel was filled with 20.0 mg/ml I solution. The exposure time was 1 min.

**Figure 7:**
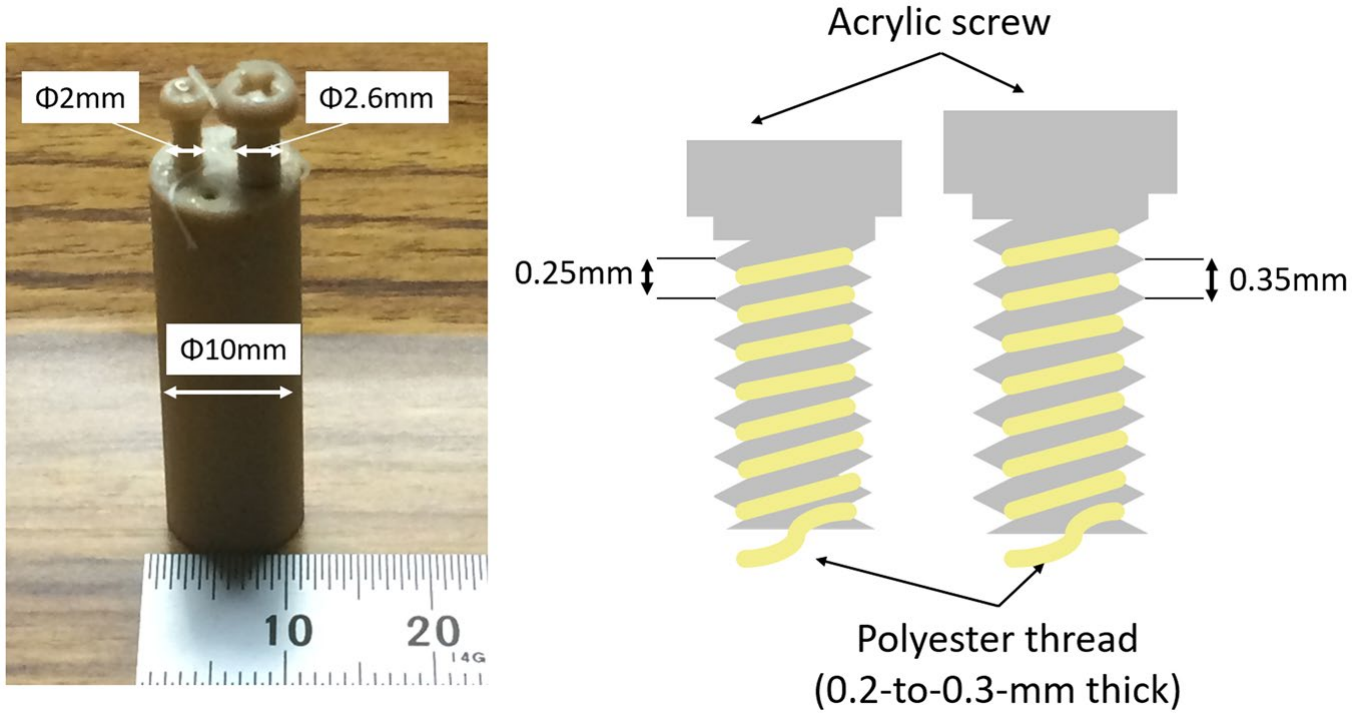
Photograph of physical phantom for spatial resolution evaluation (Phantom (IV)) with the schematic drawing of the side view, consisting of two acrylic screws along whose grooves 200–300-μm-thick Polyester thread was wound after having been dipped in I solution and allowed to dry out.

The fourth phantom (Phantom (IV)), which was employed to evaluate the 3-D spatial resolution, included two acrylic screws along whose grooves 200–300-μm-thick Polyester thread was wound after having been dipped in 20.0 mg/ml I solution and then dried out (Fig. 7). The pitch between adjacent grooves was about 0.25 mm and 0.35 mm for the small and large screws, respectively. The exposure time was 1 min.

### Reconstruction

We solved Eq. (3) using the ordered subset expectation maximization method, which is an accelerated version of ML-EM^29^. The algorithm was implemented in C# on a PC with Windows 8, Intel Xeon CPU E5-2620, and 192 GB of memory. We set the numbers of subsets and iterations to 3 and 5, respectively. The voxel size was 70 × 70 × 40, and each voxel was 0.172 mm long on each side, which is the same as the dimensions of the pixels of the Pilatus detector. The calculation times required for reconstruction were about 30 min and 240 min in the single- and multi-pinhole cases, respectively.

## Results

**Figure 8:**
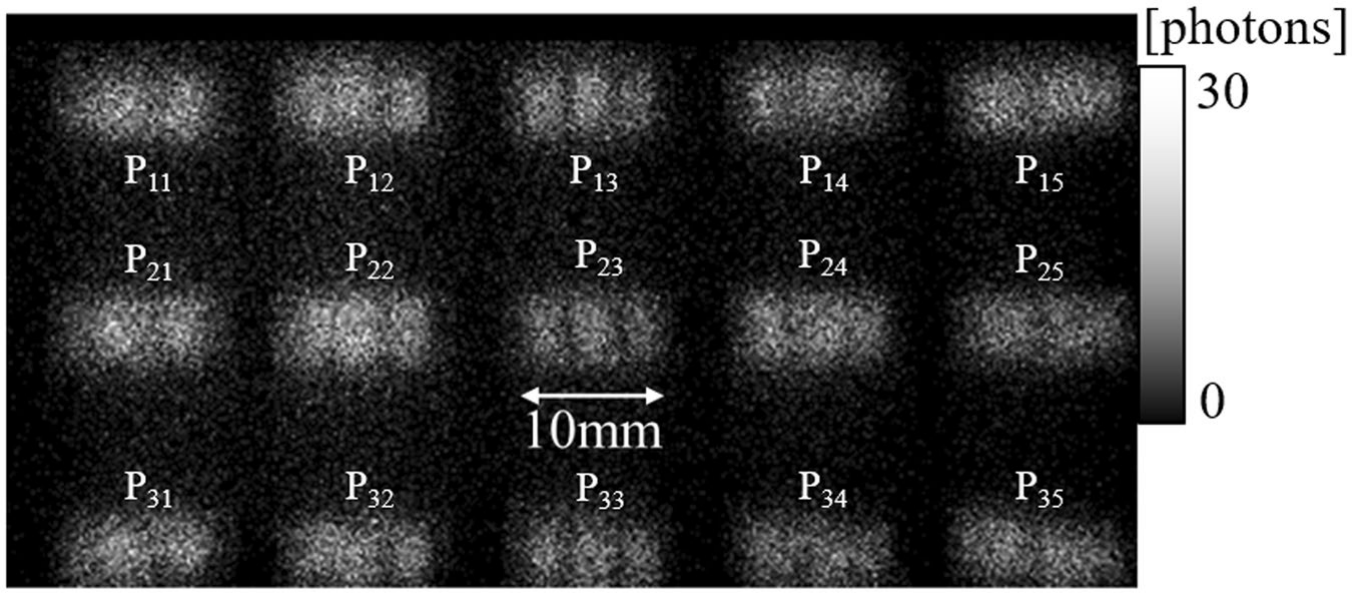
Projections of Phantom (I).

**Figure 9:**
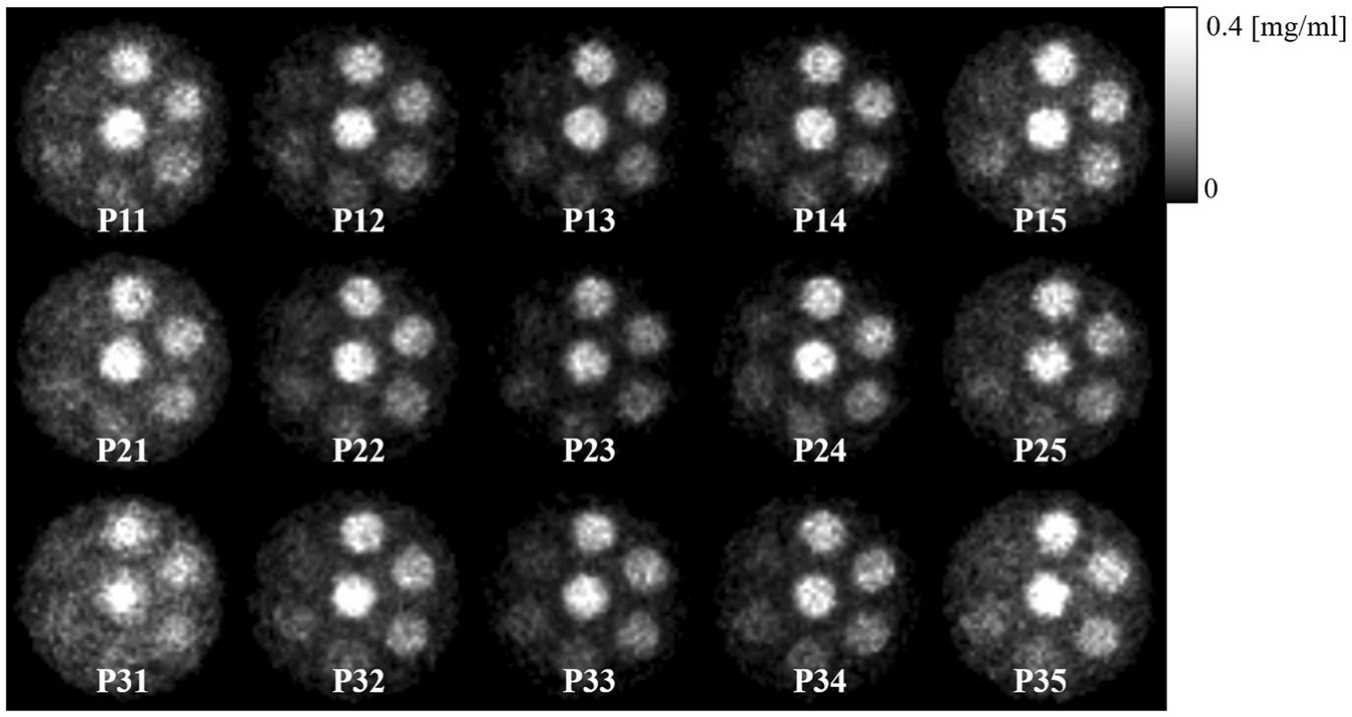
Reconstructed images of Phantom (I) acquired using individual pinholes.

**Figure 10:**
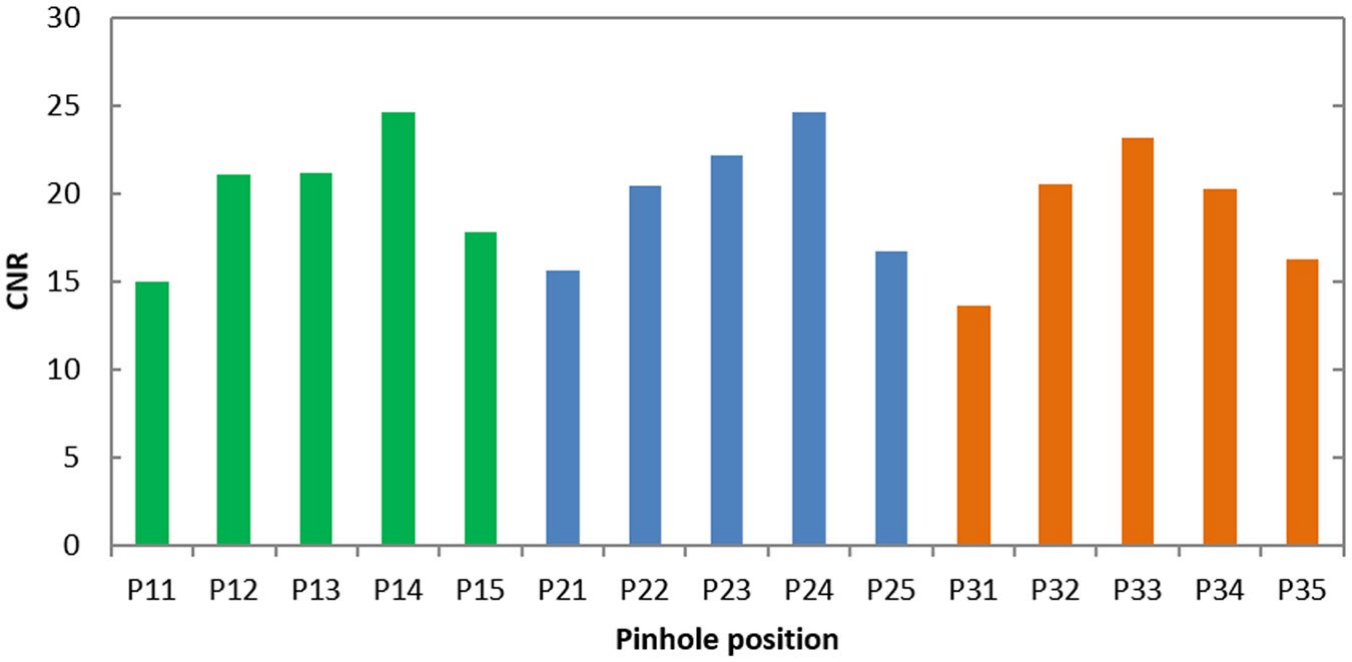
CNRs of reconstructed images of Phantom (I).

Figure 8 depicts the projections of Phantom (I) that were obtained with an exposure time of 4 min. The channels corresponding to the individual pinholes are obviously separated, and the images do not overlap. Figure 9 presents images that were reconstructed from data acquired using the individual pinholes and an exposure time of 4 min. The image from each pinhole clearly depicts the channels filled with I solutions of different concentrations. However, the image quality seems to depend on the pinhole position. So, we compared the contrast-to-noise ratios (CNRs) of the reconstructed images. The CNR was defined as

4 $$CNR=\frac{{m}_{I}-{m}_{A}}{{\sigma }_{A}},$$

where *m*_*I*_ and *m*_*A*_ are the means of the 1 × 1 × 1 mm voxel values in the 0.4 mg/ml and acrylic regions, respectively, and *σ*_*A*_ is the standard deviation in the acrylic region. The CNR was calculated for each image, and the results are presented in Fig. 10, which demonstrates that the CNR depends on the pinhole position. We will consider the cause in the subsequent section.

**Figure 11:**
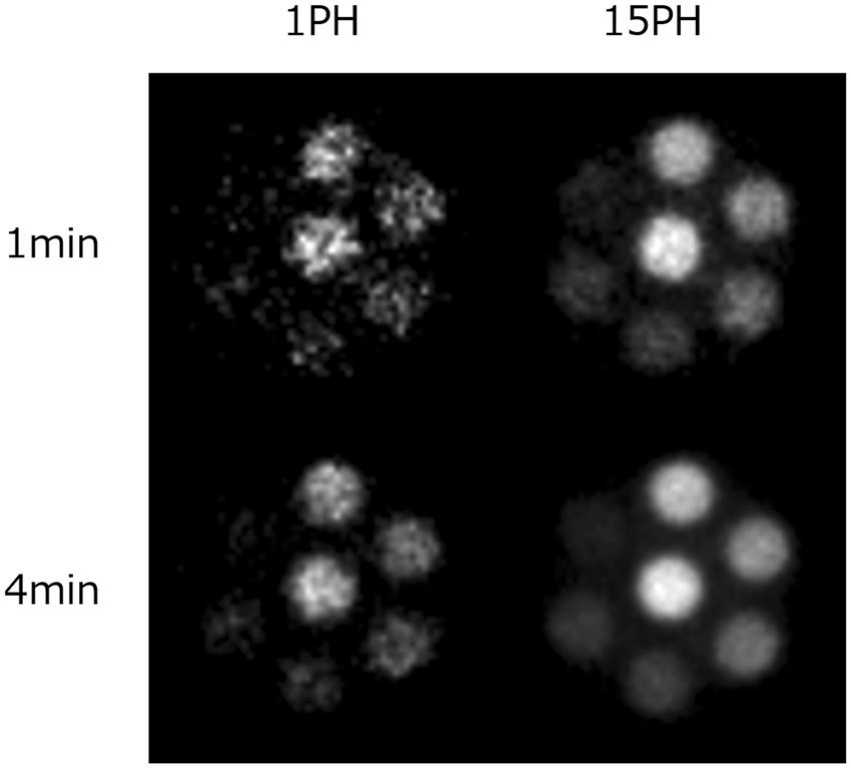
Reconstructed images of Phantom (I) obtained using exposure times of (upper) 1 min and (lower) 4 min and employing (left) one pinhole and (right) 15 pinholes.

Next, we verified the effect of the exposure time on the image quality. The upper and lower rows in Fig. 11 show the images reconstructed using exposure times of 1 min and 4 min, respectively, while the images in the left and right columns were obtained using only P_23_ and all 15 pinholes, respectively. Using a longer exposure time and multiple pinholes evidently improves the image quality. The quantitative evaluation will be discussed below. It can be concluded from the above results that the mp-FXCT system operated as intended.

**Figure 12:**
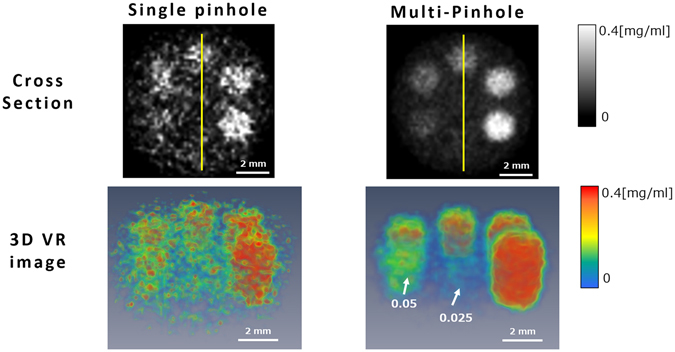
Reconstructed images of Phantom (II) acquired using (left) one pinhole and (right) 15 pinholes. The upper and lower rows present the cross-sections and 3-D images, respectively.

Figure 12 depicts cross-sections acquired at the central levels of the 3-D reconstructed images of Phantom (II) and their 3-D volume rendering (VR) images, where the images in the left and right columns were reconstructed using only P_23_ and all 15 pinholes, respectively. The background is noisier and the I-containing regions are less smooth in the single-pinhole case than in the multi-pinhole case. The CNRs in the single- and multi-pinhole cases were calculated to be 7.2 and 21.3, respectively, indicating an improvement by a factor of about three in the multi-pinhole case. On the other hand, we expect an improvement of the CNR by a factor of *N* ^1/2^ using the use of multi-pinhole, where *N* is the number of pinholes. In our case, the factor was 15^1/2^ = 3.87, which was broadly comparable to that obtained in the experiment, although it is slightly higher. This perspective would be a yardstick for the multi-pinhole design.

**Figure 13:**
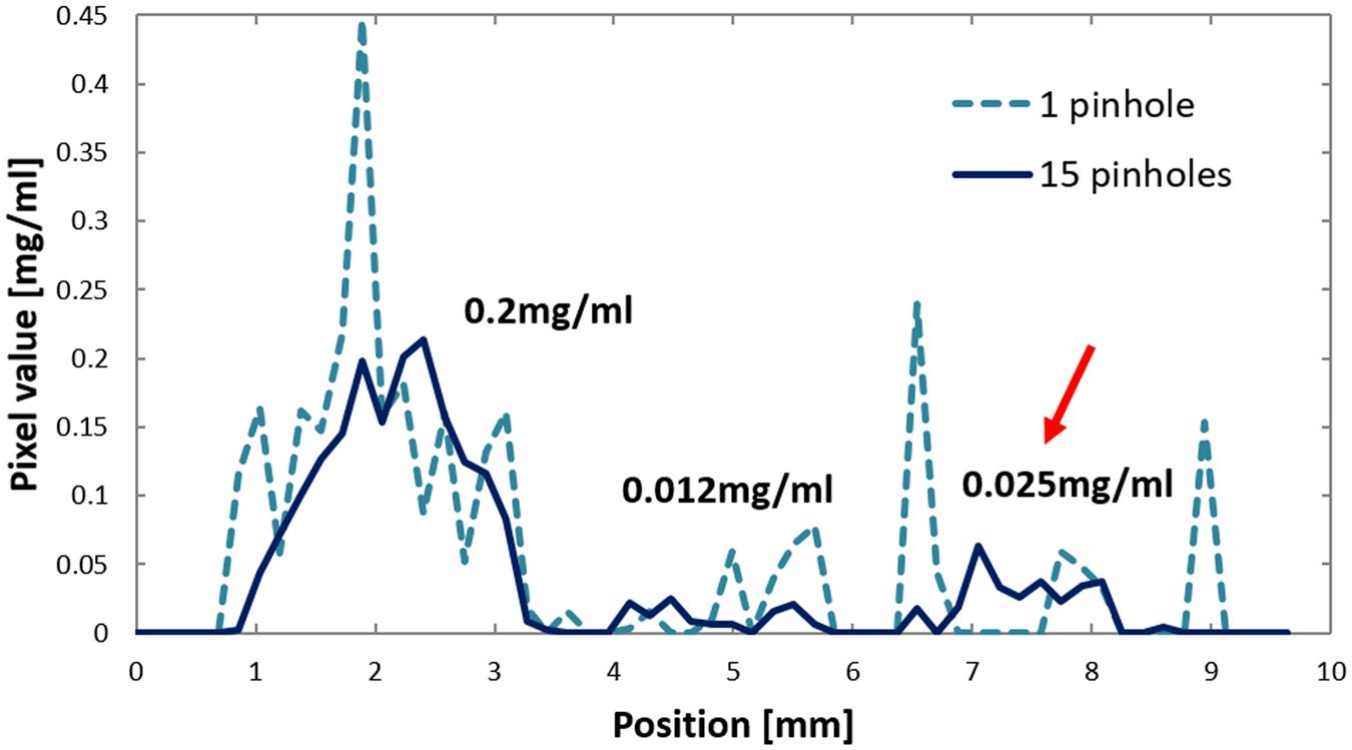
Voxel values along the yellow line in Fig. 12.

**Figure 14:**
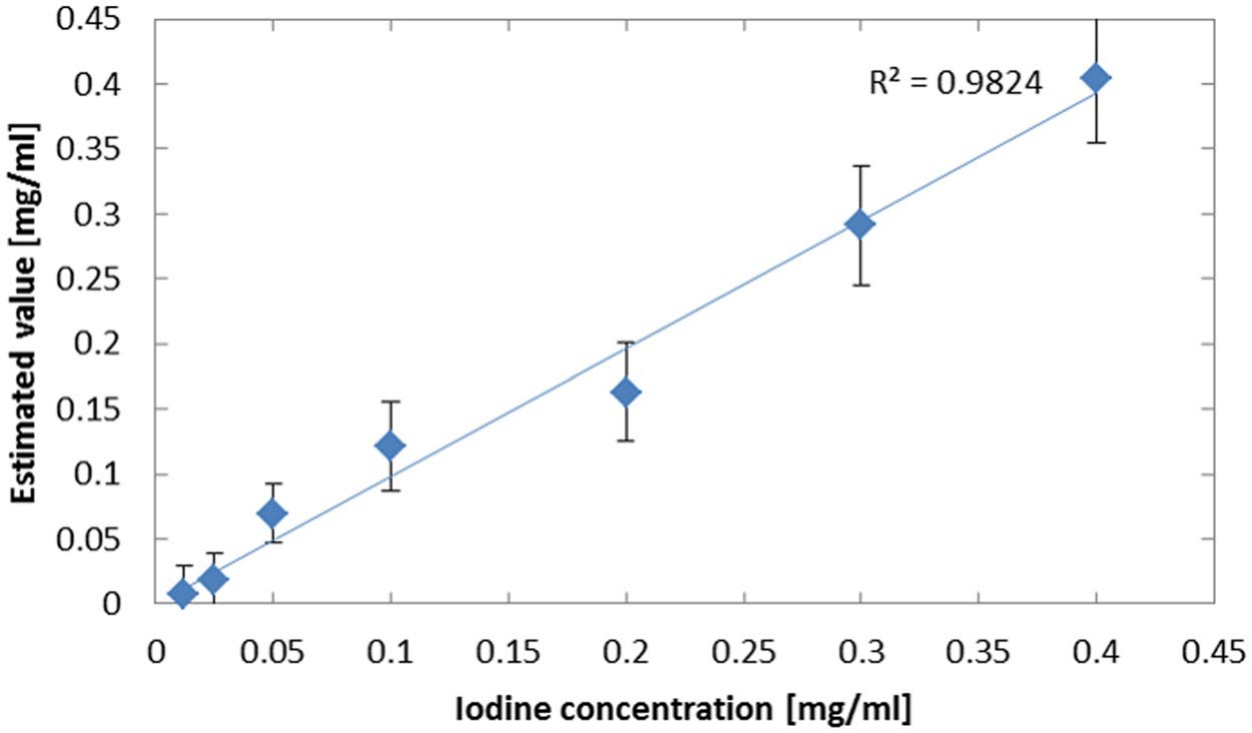
Relationship between the I concentrations estimated using mp-FXCT and the actual I concentrations.

Since the 0.012 mg/ml and 0.025 mg/ml regions are not clearly visible in the cross-sections or 3-D VR images, even in the multi-pinhole case, we compared the profiles obtained along the yellow lines in Fig. 12 that pass through those regions, which are presented in Fig. 13. Although the signals from the 0.012 mg/ml and 0.025 mg/ml regions are buried in background noise in the single-pinhole case, they seem slightly higher than the background noise in the multi-pinhole case. For more precise analysis, we calculated the LOD using the following equation^30^:

5 $$LOD={s}_{b}+3.29{\sigma }_{b},$$

where *s*_*b*_ and *σ*_*b*_ are the average and standard deviation in the background region. Choosing volumes of interest (VOIs) in the backgrounds of the 3-D reconstructed images, we calculated the LODs in the single- and multi-pinhole cases to be 0.16 mg/ml and 0.038 mg/ml, respectively. Therefore, the LOD in the multi-pinhole case was about 4.2 times higher than that in the single-pinhole case, although the hypothesis that an LOD of 0.025 mg/ml would be visible was unfortunately rejected. In addition, we investigated the quantifiability of mp-FXCT by plotting the average estimated I concentrations in VOIs set in the individual I regions against the actual I concentrations, as shown in Fig. 14. The relationship between the estimated and actual values is sufficiently linear.

**Figure 15:**
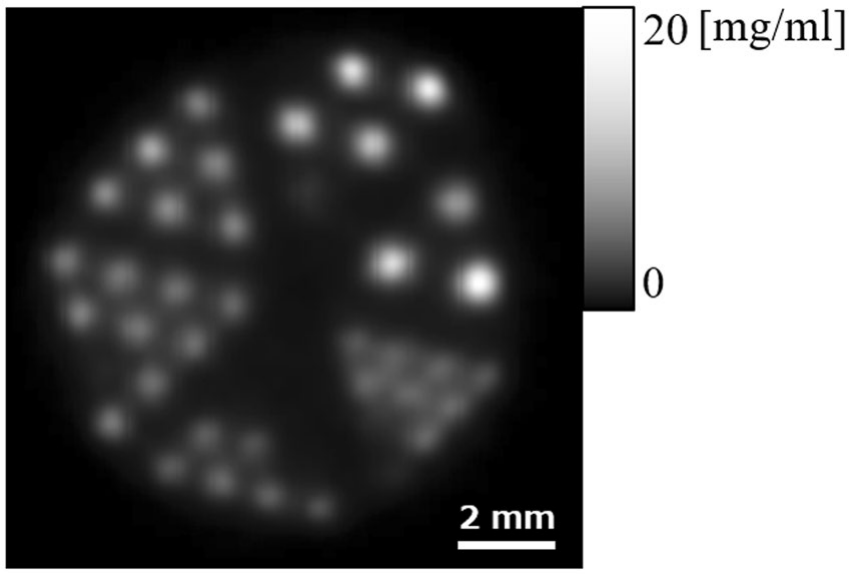
Reconstructed cross-sectional image of Phantom (III).

Figure 15 shows a cross-section obtained at the central level of the 3-D reconstructed image of Phantom (III), in which some channels are invisible because they contained air bubbles and therefore did not contain I in the central plane. Nonetheless, the region containing 0.35-mm-diameter channels is clearly visible. Thus, the horizontal spatial resolution of the mp-FXCT system is less than 0.35 mm.

**Figure 16:**
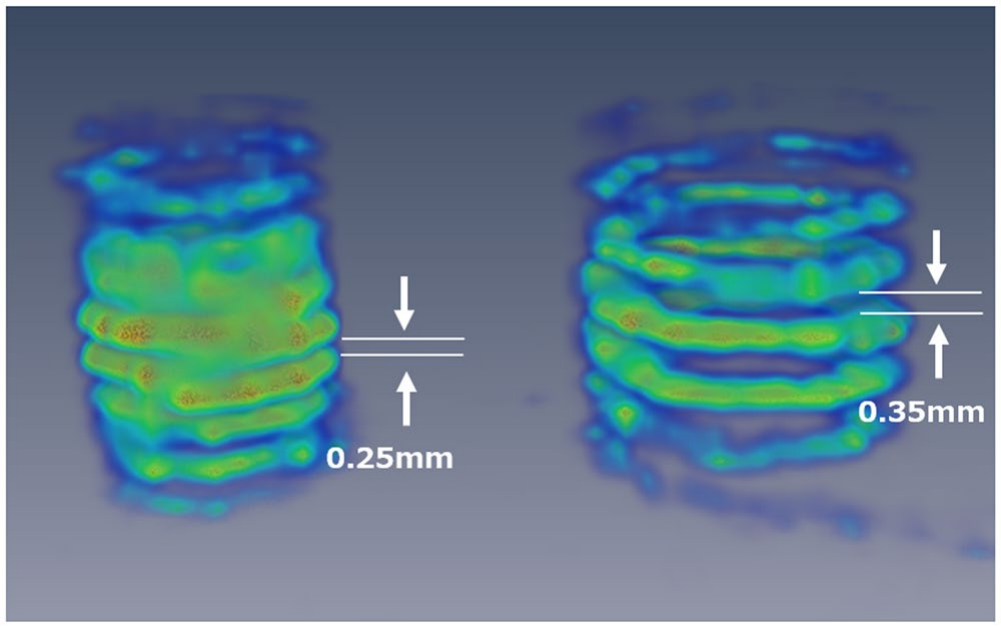
3-D image of Phantom (IV).

**Figure 17:**
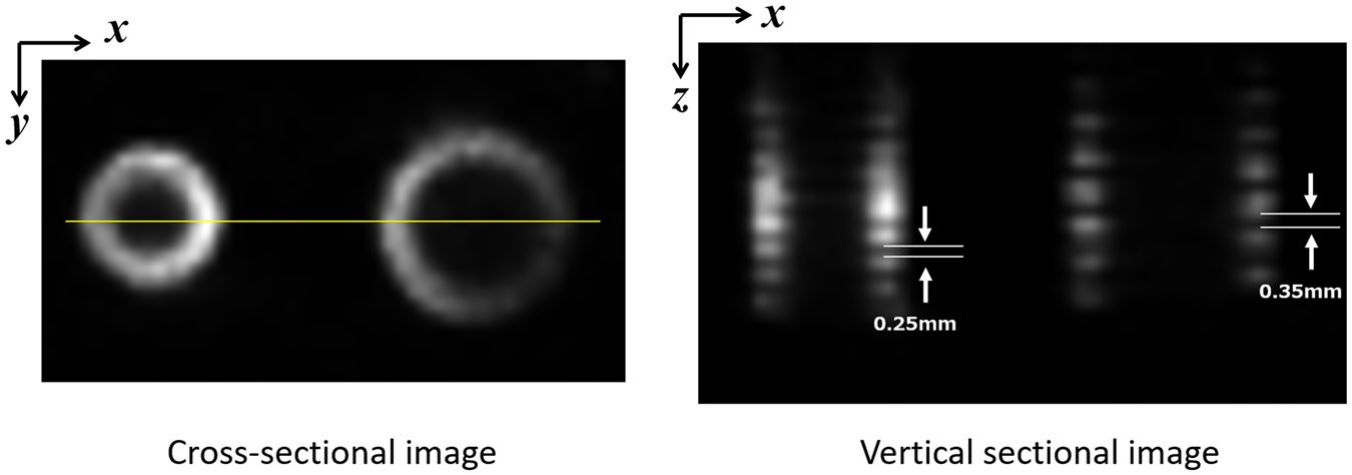
Horizontal and vertical sectional images of Phantom (IV).

Figure 16 is a 3-D VR representation of Phantom (IV). Figure 17 shows the horizontal and vertical sectional images of the reconstructed image. Although the 0.35 mm pitch of the large screw is discernible, the 0.25 mm pitch of the small screw is blurred. Thus, it can be concluded that the vertical spatial resolution of the mp-FXCT system is between 0.25 mm and 0.35 mm.

## Discussion

### Feasibility of 3-D *in-vivo* imaging

We previously developed an FXCT system in which projection data was acquired using a thin incident beam and a detector having an energy resolution sufficient to differentiate fluorescent photons from scattered photons and employed it to obtain 2-D *in-vivo* cross-sections of a mouse brain^16^. In that experiment, for a mouse anesthetized with pentobarbital and intravenously injected with ^127^I-IMP, the thalamus and hippocampus containing 0.03–0.05 mg/ml of ^127^I-IMP were delineated with a spatial resolution of about 0.5 mm in the total data acquisition time of 90 min^16^. On the other hand, the LOD and spatial resolution obtained using the mp-FXCT method were 0.038 mg/ml and about 0.3 mm, respectively, and the total measurement time was about 90 min (1 min per view). These results indicate that mp-FXCT will be effective for *in-vivo* imaging. Moreover, mp-FXCT can yield 3-D tomographic *in-vivo* images, although conventional FXCT essentially provides no more than 2-D CT scans. Thus, mp-FXCT shows promise as a potential preclinical molecular imaging modality.

Notably, the quantum efficiency of the Pilatus 100 K detector used in this experiment is only 10% near 30 keV, while the nominal quantum efficiency of the recently developed Pilatus3 X CdTe 2 M detector is 80% near 30 keV^31^. Therefore, the exposure time with this detector would be approximately eight times shorter than the current exposure time. In addition, Pilatus3 X CdTe 2 M has a sensitive area of 253.7 × 288.8 mm^2^, about 26 times wider than the 83.8 × 33.5 mm^2^ sensitive area of Pilatus 100 K. Therefore, the number of pinholes could also be increased by a factor of at least 20 by using a Pilatus3 X CdTe 2 M detector.

### Scatter reduction

In Fig. 9, the background regions of P_11_, P_21_, P_31_, P_15_, P_25_, and P_35_ are noisier than those of the other pinholes. This observation is quantitatively supported by the CNR results in Fig. 10 and can be explained in terms of x-ray scattering, primarily Compton scattering, as follows. The imaging experiment was conducted at beamline AR-NE7A, whose incident beam is linearly polarized in the horizontal plane. In theory, no Compton scattering occurs at right angles to horizontally polarized beams^6^. Since the multi-pinhole collimator was set so that the *z*-axis passed through the centre of P_23_ in our setup, no Compton scattered photons were theoretically emitted toward P_23_. In contrast, the angles between the incident beam, *i.e*., the *x*-axis, and the lines connecting the origin and the centres of the outer pinholes such as P_11_, P_21_, P_31_, P_15_, P_25_, and P_35_ differed more than the corresponding angles for the inner pinholes from right angles. Accordingly, the contamination due to scattered photons was more significant for the outer pinholes than for the inner pinholes. In the case that the number of pinholes is increased by employing a detector having a wider sensitive area, the contamination due to scattered radiation gets worse. The problem would be overcome by the dual-energy measurement which will be discussed below.

In addition, although we installed a Sn filter to prevent scatter contamination in front of the multi-pinhole collimator, the experimental results demonstrate that the filtering was insufficient since scatter contamination degraded the reconstructed image quality. To circumvent this problem, dual-energy data acquisition, in which two sets of projections are acquired at incident energies just above and below the K-edge of the imaging agent and 3-D images are reconstructed from the projections based on a statistical model incorporating the scatter components, could be employed^22^. Although dual-energy data acquisition would require twice the measurement time if the same detector were used, that issue could be overcome by replacing the current detector with the state-of-the-art detector and increasing the number of pinholes.

### Radiation dose

When a water cylinder of *r* = 20.0 [mm] in radius is irradiate with a parallel monochromatic x-ray beam which is perpendicular to the axis and covers the sample width, the theoretical radiation absorbed dose is given as^32^.

6 $$D=\frac{2}{\pi r\rho }{E}_{abs}(1-g)T[{\rm{J}}\,{\rm{k}}{{\rm{g}}}^{-1}],$$

where *ρ* [kg m^−3^] and *T* [s] are the density of water and the exposure time, respectively;

$$\begin{array}{rcl}{E}_{abs} & = & \varphi {E}_{x \mbox{-} ray}({\mu }_{E}/{\mu }_{A})[{\rm{J}}\,{{\rm{m}}}^{-2}{{\rm{s}}}^{-1}],\\ g & = & {\int }_{0}^{\frac{\pi }{2}}\exp (-2r{\mu }_{A}\,\cos \,\theta )\,\cos \,\theta \,d\theta \end{array}$$

where *E*_x*-ray*_ [J], *ϕ* [m^−2^ s^−1^], *μ*_*A*_ [m^−1^], and *μ*_*A*_ [m^−1^] are the x-ray energy, the fluence rate of the incident beam in front of the sample, the linear attenuation coefficient of water at *E*_x*-ray*_, and the linear energy absorption coefficient of water at *E*_x*-ray*_, respectively^33^. Here, we neglect the effect of secondary radiation for simplicity. When *E*_x*-ray*_ = 33.4 [keV] = 5.34 × 10^−15^ [J], *ϕ* = 9.3 × 10^7^ [mm^−2^ s^−1^] = 9.3 × 10^13^ [m^−2^ s^−1^], *μ*_*A*_ = 30.5 [m^−1^], *μ*_*E*_ = 9.88 [m^−1^], *ρ* = 1.0 × 10^3^ [kg m^−3^], *T* = 60 [s], and *r* = 0.02 [m], we obtained *D* = 8.13 × 10^−2^ [Gy] from Eq. (5), in which *g* (=0.736) was numerically calculated. When the number of projections is 90, the total radiation dose is 8.13 × 10^−2^ × 90 = 7.31 [Gy]. A generally known value of LD50 (50% lethal dose) for mice is about 7 Gy^34^. Thus, the estimated value is somewhat large. From Eq. (6), the easiest way to reduce the absorbed dose is to shorten the exposure time *T*. As mentioned above, the quantum efficiency of the Pilatus 100 K detector is 10% near 30 keV. In contrast, that of Pilatus3 X CdTe 2 M detector is 80%. Therefore, the exposure will be shortened by a factor of at least 8. Another way to reduce the absorbed dose is to increase the number of pinholes. As discussed previously, if we use a wider sensitive area detector, we can easily increase the number of pinholes. In addition, the means of reducing the exposure time would be to use two detectors. In our imaging system, the pinhole collimator and detector system were located on only one side of the incident beam, so the space on the opposite side was free. If two detectors were to be used, the exposure time could be halved. Moreover, if free space existed above or below the sample, additional detectors could be employed.

## Conclusion

We proposed an mp-FXCT technique for fast data acquisition. The experimental results obtained using physical phantoms demonstrated that mp-FXCT enables 3-D imaging of 15 × 15 × 5 mm^3^ subjects with high quantifiability, a spatial resolution less than 0.35 mm, and a low LOD of 38 μg/ml. The imaging properties were almost identical to those of conventional FXCT when used to perform data acquisition with a thin beam to obtain *in*-*vivo* 2-D cross-sectional images of a mouse brain. Therefore, mp-FXCT will pave the way for 3-D *in-vivo* imaging of small animals using non-radioactive imaging agents, *i.e*., preclinical molecular imaging. By using a state-of-the-art detector, multi-fold improvements in the spatial resolution and LOD can be realized. *In-vivo* imaging experiments using mice will be the focus of our work in the immediate future.
